# Long Noncoding RNAs in the Regulation of Asthma: Current Research and Clinical Implications

**DOI:** 10.3389/fphar.2020.532849

**Published:** 2020-09-11

**Authors:** Xueyi Zhu, Ying Wei, Jingcheng Dong

**Affiliations:** ^1^Department of Integrative Medicine, Huashan Hospital, Fudan University, Shanghai, China; ^2^Institutes of Integrative Medicine, Fudan University, Shanghai, China

**Keywords:** asthma, long noncoding RNAs, airway inflammation, airway remodeling, glucocorticoid insensitivity

## Abstract

Asthma is a chronic airway inflammatory disorder related to variable expiratory airflow limitation, leading to wheeze, shortness of breath, chest tightness, and cough. Its characteristic features include airway inflammation, airway remodeling and airway hyperresponsiveness. The pathogenesis of asthma remains extremely complicated and the detailed mechanisms are not clarified. Long noncoding RNAs (lncRNAs) have been reported to play a prominent role in asthma and function as modulators of various aspects in pathological progress of asthma. Here, we summarize recent advances of lncRNAs in asthma pathogenesis to guide future researches, clinical treatment and drug development, including their regulatory functions in the T helper (Th) 1/Th2 imbalance, Th17/T regulatory (Treg) imbalance, eosinophils dysfunction, macrophage polarization, airway smooth muscle cells proliferation, and glucocorticoid insensitivity.

## Introduction

Asthma is a common pulmonary disease, featured by airway inflammatory response, airway hyperresponsiveness (AHR), and airway remodeling. The increasing incidence of asthma has caused a huge economic burden on a global scale. The epidemiological studies conducted by the Center for Disease Control have proposed that approximately $56 billion was used in asthma costing in the United States in 2007 (Loftus and Wise, 2015). Previous studies (Mitchell and O’Byrne, 2017; Tumes et al., 2017) have mostly focused on the involvement of lymphocyte subsets, eosinophils (EOS), neutrophils, macrophages, etc., as well as their cytokines and chemokines in asthma pathogenesis. Besides, recent studies (Kapranov et al., 2005; Corner et al., 2015; Marques-Rocha et al., 2015; Narozna et al., 2017) have shown that long noncoding RNAs (lncRNAs), used to be considered useless, definitely play a crucial role in the regulation of asthma and can act as novel therapeutic targets.

**Figure 1:**
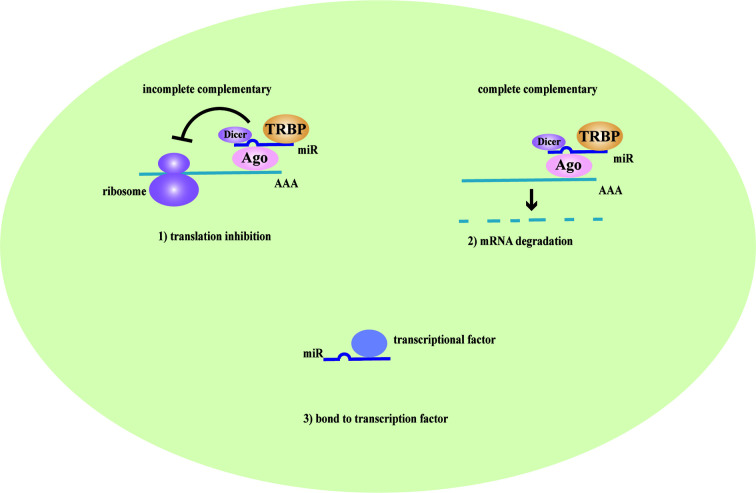
The mechanism of miRs. MicroRNAs (MiRs) are bond with argonaute proteins (Ago), Dicer and trans-activation response RNA-binding protein (TRBP) to form the RNA-induced silencing complex (RISC). In the case of incomplete complementary sequences, they bind to mRNA to translation inhibition. In the case of complete complementary sequences, they bind to 3’ untranslated regions (UTR) of mRNA to mRNA degradation. Besides, they can bind to transcription factors to coregulate the transcription of genes.

**Figure 2:**
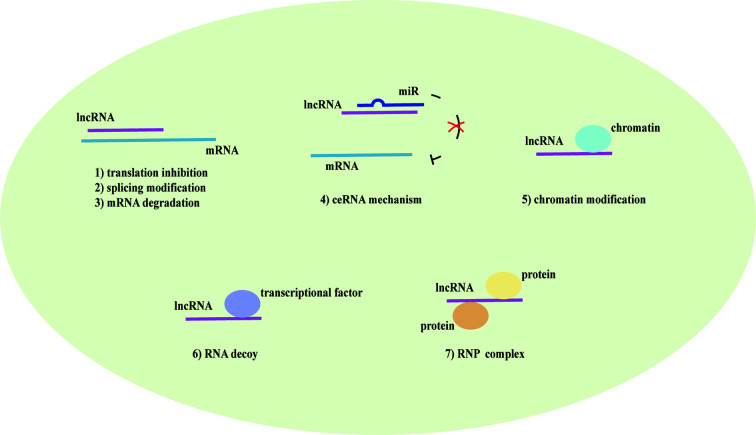
The mechanism of lncRNAs. Long noncoding RNAs (lncRNAs) direct bind to the genes to translation inhibition, splicing modification, and mRNA degradation. Alternatively, they act as a competitive endogenous RNA (ceRNA) that has the microRNA (miR) response element to sponge the miR to further prevent mRNA degradation and enhance the mRNA expression. lncRNAs also mediate chromatin remodeling for chromatin modification and function as an RNA decoy that binds to the transcription factors to regulate the downstream signaling pathways. They assemble ribonucleoprotein (RNP) complexes to modulate protein stability as well.

LncRNAs are non-protein coding transcripts with a length of at least 200 nucleotides (Hung and Chang, 2010) and are generally classified into antisense lncRNAs, pseudogenes lncRNAs, intronic lncRNAs, and intergenic lncRNAs by protein-coding gene sequences and position (Booton and Lindsay, 2014). Referring to their classical mechanisms, they can not only regulate genes (translation inhibition, splicing modification, and mRNA degradation) *via* the direct bond to the genes but also sponge the microRNA (miR) as a competitive endogenous RNA (ceRNA) to prevent mRNA degradation and stabilize the mRNA (Sun et al., 2018; Svitich et al., 2018; Sun et al., 2019). Most lncRNAs with low abundance and instability only target adjacent chromatin regions, which are called cis-acting lncRNAs. Alternatively, a few lncRNAs with high abundance and stability migrate and target the distant chromatin regions, which are called trans-acting lncRNAs (Rinn and Chang, 2020). Recent studies have proposed that lncRNAs and the target genes share the same miR response element and competitively bind to the miRs, thereby preventing mRNA degradation by miR and increasing mRNA expression. MiRs are noncoding single-strand RNAs with a length of fewer than 21 nucleotides. They are known to have the ability to target genes at both transcriptional and posttranscriptional levels, including translation inhibition, mRNA degradation and transcription factor modulation (Mousavi et al., 2019) (**Figure 1**). In the nucleus, primary miRs (pri-miRs) are transcribed by RNA polymerase (pol) II or III. Then, Drosha-DiGeorge critical region 8 (DGCR8) compounds cleavage them into precursor miRs (pre-miRs), which are in the shape of a hairpin. Subsequently, Exportin-5-Ran-GTP compounds export them to the cytoplasm. In the cytoplasm, Dicer combined with trans-activation response RNA-binding protein (TRBP) cleavage them into mature miR duplex, which is loaded into argonaute (Ago) 2 protein to form the RNA-induced silencing complex (RISC). In a manner of incomplete complementary sequences, miRs can bind to 3’ untranslated regions (UTR) of mRNAs to suppress the ribosome for protein translation. In a manner of complete complementary sequences, miRs bind to 3’ UTR of mRNAs for mRNA degradation (Bartel, 2009). Another mechanism of lncRNAs is chromatin modification. They form chromatin remodeling complexes to modulate ubiquitination and methylation. Moreover, they are as similar to DNA, which can function as an RNA decoy that binds to the transcription factors to regulate their activity and downstream signaling pathways. They also assemble the ribonucleoprotein (RNP) complexes to regulate the stability of proteins (Quinn and Chang, 2016) (**Figure 2**).

**Figure 3:**
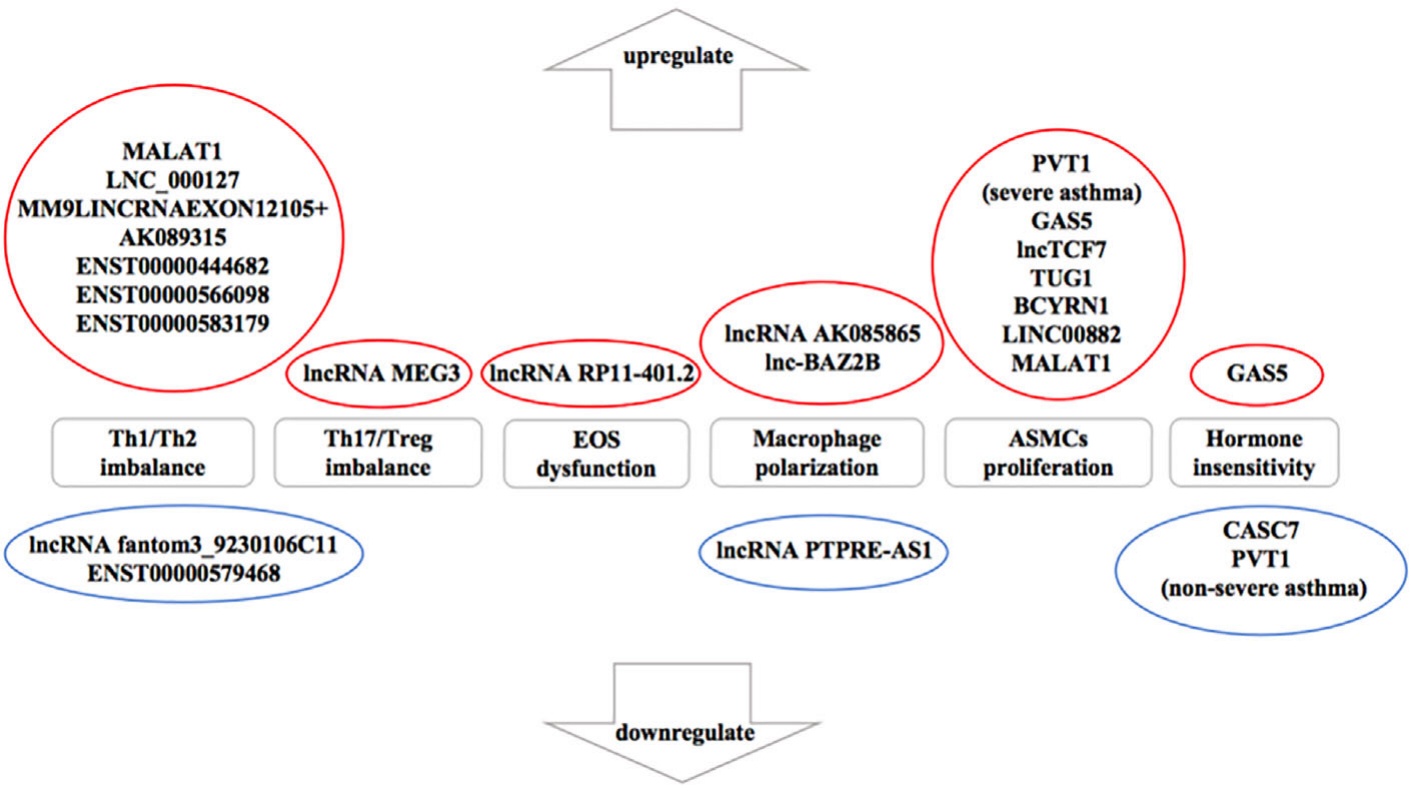
The influence of downregulated and upregulated lncRNAs in asthma pathogenesis. Blue indicates the inhibition function and red indicates the promotion function. According to the current studies of asthma, seven long noncoding RNAs (lncRNAs) are upregulated and two lncRNAs are downregulated in T helper (Th) 1/Th2 imbalance. lncRNA MEG3 is upregulated in Th17/T regulatory (Treg) imbalance. In addition, lncRNA RP11-401.2 is upregulated in EOS dysfunction and seven lncRNAs are upregulated in airway smooth muscle cells (ASMCs) proliferation. However, previous studies have not yet found the downregulated lncRNAs in Th17/Treg imbalance, EOS dysfunction or ASMCs proliferation. lncRNA AK085865 and lnc-BAZ2B are upregulated whereas lncRNA PTPRE-AS1 is downregulated in macrophage polarization. On the aspect of glucocorticoid insensitivity in asthma, lncRNA growth arrest-special transcript 5 (GAS5) is upregulated whereas cancer susceptibility candidate 7 (CASC7) and lncRNA plasmacytoma variant translocation (PVT1) (nonsevere asthma) are downregulated.

Numerous studies (Ezegbunam and Foronjy, 2018; Liu X. et al., 2019) have described that lncRNAs participate in the progress of asthma that mediate multiple signaling pathways and act as biomarkers for the phenotypes of asthma as well as regulators of airway inflammation, remodeling, and glucocorticoid sensitivity. It was proved that the expression of lncRNAs showed remarkable differences in peripheral blood between therapy-resistant asthmatic children and controlled asthmatic children (Persson et al., 2015). LncRNAs also modulated T cell functions *via* regulating mRNAs in asthma (Tsitsiou et al., 2012). Here, we review established literature on lncRNAs in asthma (**Table 1**) and discuss their regulation of T helper (Th) 1/Th2 imbalance, Th17/T regulatory (Treg) imbalance, EOS dysfunction, macrophage polarization, airway smooth muscle cells (ASMCs) proliferation and glucocorticoid insensitivity to further clarify the specific mechanisms of lncRNAs in asthma pathogenesis (**Figure 3**).

**Table 1:** Dysregulated lnRNAs in asthma pathogenesis.

lnRNA	Target	Tissue/Cell	Intervention	Expression	Mechanism	Signaling pathways	Clinical	Ref.
Malat1	miR-155, miR-150	peripheral blood, ASMCs, CD4+T cells (human)	/	upregulate	sponge miR-155 and hinder its bond with CTLA-4 to break Th1/Th2 balance, act as a ceRNA for miR-150 and induce ASMCs proliferation	MALAT1/miR-155/CTLA-4 axis, eIF4E/Akt signaling	act as markers in dysregulated Th1/Th2 imbalance and modulate airway remodeling in asthma	(Liang and Tang, 2020), (Lin L. et al., 2019)
LNC_000127	/	CD4^+^T cells (human), Jurkat cells	/	upregulate	promote Th2 inflammation in eosinophilic asthma	TCR/STAT/GATA3 pathway	distinguish the phenotype of eosinophilic asthma	(Zhu et al., 2019)
lncRNA fantom3_9230106C11	GATA1, miR-19	CD4^+^T cells (mouse)	/	downregulate	associated with Th2 cell differentiation	/	mediate Th2 cell differentiation and regulate type 2 inflammation	(Wang et al., 2019)
MM9LINCRNAEXON12105+ and AK089315	/	memory T cells (mouse)	iPSC- MSCs	upregulate (without iPSC- MSCs), downregulate (iPSC- MSCs)	involved in the Th2-type immune response	/	induce type 2 inflammation	(Wang et al., 2017)
ENST00000444682	SMAD7, WNT2B, C/EBP, T-bet, and NF-kB	CD4^+^T cells, isolated from peripheral blood(human)	/	upregulate	positively correlated with IL-13 and IL-5, and negatively correlated with IL-6	SMAD7 cAMP/C/EBP/T-bet/NF-kB	modulate Th2 cell differentiation and the related proinflammatory factor production	(Qi et al., 2018)
ENST00000566098				upregulate	positively correlated with IL-13			
ENST00000583179				upregulate	positively correlated with IL-13 and negatively correlated with IL-4 and IL-6			
ENST00000579468				downregulate	positively correlated with IL-5 and IL-13			
lncRNA MEG3	miR-17	CD4^+^T cells isolated from peripheral blood mononuclear cells (human)	/	upregulate	act as a ceRNA to sponge miR-17, thus repressing the bond of miR-17 to RORγt to prevent RORγt mRNA degradation	miR-17/RORγt	regulate the balance of Treg/Th17	(Qiu et al., 2019)
lncRNA RP11-401.2	/	peripheral blood and sputum(human)	/	upregulate	promote EOS dysfunction	/	regulate eosinophilic asthma	(Zhu et al., 2018)
lncRNA AK085865	/	macrophages (mouse)	/	upregulate	promote M2 macrophage polarization and enhance ILC2 differentiation (amplify type 2 inflammation)	/	regulate M2 macrophages and ILC2s	(Pei et al., 2020)
lnc-BAZ2B	BAZ2B	peripheral blood (human), macrophage (mouse)	/	upregulate	increase BAZ2B to enhance IRF4 and M2 macrophage activation	/	a target for modulating type 2 inflammation	(Xia et al., 2020)
lncRNA PTPRE-AS1	WDR5	PBMCs(human), macrophage (mouse)	/	downregulate	increase PTPRE expression to suppress IL-4-induced M2 macrophages	MAPK/ERK 1/2 pathway	act as a biomarker to attenuate M2 macrophage-mediated type 2 inflammation	(Han et al., 2019)
PVT1	c-MYC	ASMCs(human)	/	upregulate (severe asthma), downregulate (nonsevere asthma)	DEX increases c-MYC, which can bind to lncRNA PVT1 to promote ASMCs proliferation in severe asthma. The knockdown of lncRNA PVT1 reverses the inhibitory effect of DEX, amplifying glucocorticoid insensitiveness	PVT1/c-MYC	distinguish ASMCs phenotype and regulate glucocorticoid sensitiveness	(Austin et al., 2017)
GAS5	miR-10a	ASMCs(mouse)	/	upregulate	promote ASMCs proliferation through GAS5/miR-10a/BDNF and reduce glucocorticoid sensitivity	GAS5/miR-10a/BDNF	regulate airway remodeling and modulate glucocorticoid sensitivity	(Zhang et al., 2018), (Keenan et al., 2015)
lncTCF7	TIMMDC1/AKT	ASMCs (human)	/	upregulate	enhance ASMCs growth and migration *via* activating the TIMMDC1/AKT signaling pathway	TIMMDC1/AKT pathway	a potential therapeutic for airway remodeling	(Fan et al., 2019)
TUG1	miR-590-5p	ASMCs (rat)	/	upregulate	sponge miR-590-5p and prevent its bond to FGF1 (promote ASMCs proliferation and migration)	TUG1/miR-590-5p/FGF1	a target for modulating airway remodeling	(Lin J. L. et al., 2019)
BCYRN1	TRPC1	ASMCs (rat)	/	upregulate	upregulate stability of TRPC1 to promote ASMCs viability, proliferation and migration	TRPC1 pathway	a target for regulating airway remodeling	(Zhang et al., 2016)
LINC00882	miR-3619-5p	ASMCs (human fetus)	/	upregulate	sponge miR-3619-5p and prevent its bond to β-catenin to enhance PDGF-induced fetal ASMCs proliferation	Wnt/β-catenin signaling	modulate ASMCs proliferation in pediatric asthma	(Perry et al., 2014), (Liu Z. F. et al., 2019)
CASC7	miR-21	ASMCs(human)	/	downregulate	sponge miR-21 and suppress its bond to PTEN, thus enhancing PTEN expression (elevate corticosteroid sensitivity)	PI3K/AKT signaling pathway	enhance glucocorticoid sensitivity	(Liu J. H. et al., 2019)

## lncRNAs Associated With Inflammation in Asthma Pathogenesis

### lncRNAs Associated With Th1/Th2 Imbalance

Asthma exacerbation is often attributed to the imbalance of Th1/Th2, that Th2 cells secrete Th2-type cytokines [interleukin (IL)-4, IL-5, and IL-13] to amplify type 2 inflammation while Th1 cells secrete Th1-type cytokines [interferon (IFN)-γ, IL-2, lymphotoxin (LT)-α, and tumor necrosis factor (TNF)-α] to confine type 2 inflammation, but mediate type 1 inflammation (Foster et al., 2017; Mukherjee and Nair, 2018). Th2 cells and type 2 pulmonary innate lymphoid cells (ILC2s) are dominant in type 2 inflammation to modulate eosinophilic asthma. Th1 cells are closely related to autoimmune and have antiinflammatory ability but if they are highly expressed, it will probably result in neutrophilic inflammation or severe asthma. Despite the glucocorticoid therapies have a great effect on maintaining the balance of Th1/Th2 in asthma, more studies have paid attention to various treatments targeting Th2-associated IL-4, IL-5, or IL-13. It has been demonstrated that IL-5, IL-13, or IL-4 deficient mice are protected from the inflammation and the damage of the lung (Foster et al., 1996; Hogan et al., 1997; Mattes et al., 2002). Furthermore, this kind of therapy can attenuate the pulmonary inflammation and enhance the lung function of the asthmatic patients (Wenzel et al., 2007; Corren et al., 2011; Krug et al., 2015). Several lncRNAs have been revealed to act as a modulator of Th1 and Th2 inflammation in asthma. Li et al. (2020) confirmed that lncRNA NEAT1 was upregulated and were negatively correlated with miR-124. Also, it was positively correlated with Th1-associated TNF-α, IL-1β and Th17-associated IL-17 to enhance the exacerbation risk of asthma, which showed its potential to regulate Th1 inflammation in asthma. Hence, we describe the role of these lncRNAs in Th1/Th2 balance specifically.

#### Metastasis-Associated Lung Adenocarcinoma Transcript 1 (MALAT1)

Liang and Tang (2020) found that MALAT1 was upregulated and miR-155 was downregulated in the blood of asthmatic patients. Th1-type cytokines (IL-2 and IFN-γ)/Th2-type cytokines (IL-4 and IL-10) ratio and Th1-type transcription factors (T-bet)/Th2-type transcription factors (GATA3) ratio dropped, which implied the attenuation of Th1 inflammation and the amplification of Th2 inflammation. After the cotransfection with the plasmids and miR-155 mimic or the plasmids and miR-155 inhibitor, it was confirmed that MALAT1 was negatively correlated with the Th1/Th2 ratios whereas miR-155 was positively correlated with these ratios. According to the luciferase reporter gene assay *in vitro*, MALAT1 was proved to sponge miR-155 to suppress its expression in CD4^+^T cells, thus breaking Th1/Th2 balance. Furthermore, miR-155 could bind with cytotoxic T-lymphocyte-associated protein (CTLA) -4 and CTLA-4 hindered miR-155 mimic-mediated or MALAT1 siRNA-mediated Th1 cell differentiation, amplifying Th1/Th2 imbalance. It can be concluded that the MALAT1/miR-155/CTLA-4 axis has the capability to modulate Th1/Th2 balance in asthma and act as markers in dysregulated inflammation in asthma.

#### LNC_000127

Zhu et al. (2019) observed 190 unique lncRNAs in eosinophilic asthmatic patients and 6851 aberrant mRNAs by RNA-sequencing (RNA-seq). The enriched pathways included the T cell receptor signaling pathway, which implied that they might get involved in T cell-mediated inflammation. Based on the construction of the mRNA-lncRNA correlation network and the choice of eosinophilic asthma-associated genes (Il5RA, GATA3, SATAT5, and SOCS), three lncRNAs (RP11-408H1.3, LNC_000127, OIP5-AS1) were focused to be coexpressed with those genes above. To further verify *via* quantitative real-time PCR (qRT-PCR), LNC_000127 was highly expressed in Eos samples while it was diminished in eosinophilic asthmatic patients who were cured *in vivo*. Phorbol 12-myristate 13-acetate (PMA)/CD_28_ used to be proved to have the capability to induce Th2-specific GATA3 and LNC_000127 was significantly enhanced in PMA/CD_28_-induced Jurkat cells. This illustrated that LNC_000127 could positively modulate the Th2 pathway. Depending on the knockdown of LNC_000127 *in vitro*, Th2-type genes (GATA3, CD40L, CCR8, STAT5A, and CRLF2) were decreased in both RT^2^ profiler™ PCR array and western blot analysis. These findings all supported that LNC_000127 was closely involved with Th2 inflammation *via* TCR/STAT/GATA3 pathway and was a biomarker for eosinophilic asthma. The mechanism of whether it can act as a ceRNA to regulate the inflammation remains to be explored in the future.

#### lncRNA fantom3_9230106C11

It was revealed that 36 lncRNAs were upregulated and 98 lncRNAs were downregulated while 160 mRNAs were upregulated and 141 mRNAs were downregulated in CD4^+^T cells of ovalbumin (OVA)-induced asthmatic mice *via* next-generation sequencing (Wang et al., 2019). Based on the constructed lncRNA-mRNA coexpression network, lncRNA fantom3_9230106C11 was highlighted to have a high degree of connectivity and its expression in CD4^+^T cells was consistent with sequencing data. It was significantly downregulated *in ex vivo* as well as in Th2 cells *in vitro* by further qRT-PCR validation, which pointed to its ability to modulate Th2 cell differentiation. Using LncTar, Bibiserv and RNA 22 analysis, lncRNA fantom3_9230106C11 was predicted to bind to transcription factor GATA1 and miR-19 to be related to Th2 cell differentiation, which tended to be further proved. The findings provide the conclusion that lncRNA fantom3_9230106C11 has the potential to bind to miRs and transcription factors to mediate Th2 cell differentiation and regulate type 2 inflammation.

#### lncRNAs MM9LINCRNAEXON12105+ and AK089315

Induced pluripotent stem cell (iPSC)-mesenchymal stem cells (MSCs) were demonstrated to have the potential to alleviate inflammation and diminish Th2-associated cytokines in asthmatic mice (Wang et al., 2017). There were 846 differently expressed lncRNAs *in vivo* and 4176 differentially expressed lncRNAs in the coculture of memory T cells from asthmatic mice and iPSC-MSCs *in vitro*. After selecting the overlapping lncRNAs, 23 lncRNAs and 96 mRNAs were gained. 58 target genes were predicted to be the potential targets of these 23 lncRNAs. Further bioinformatic analysis and Pearson’s correlation coefficient showed that 9 lncRNAs were coexpressed with these 96 mRNAs that were enriched in the immune response and inflammatory cytokines and receptors, mainly linked with Th2-associated cytokines (IL-4, IL-5 and IL-13), Th2-associated transcriptional factors (STAT5 and STAT6) and Th2-associated chemokines (CCL17 and CCL22). They all promoted Th2 cell differentiation and recruitment. LncRNAs MM9LINCRNAEXON12105+ and AK089315 were finally identified to have the best conformance and stability *via* qRT-PCR validation. These two lncRNAs might be the major therapeutic targets of iPSC-MSCs and probably linked with the regulation of Th2-type inflammation in asthma. The specific mechanism could be confirmed in the further cotransfection and the luciferase reporter gene assay.

#### ENST00000444682, ENST00000566098, ENST00000583179, and ENST00000579468

It was demonstrated that 2,725 lncRNAs and 3,167 mRNAs were differentially expressed in CD4^+^T cells between asthmatic patients and the control group by microarrays (Qi et al., 2018). Using qRT-PCR to validate these dysregulated lncRNAs, lncRNAs ENST00000444682, ENST00000566098 and ENST00000583179 were highly expressed while ENST00000579468 was lowly expressed, consistent with the microarrays results. With the further analysis of Spearman correlation, ENST00000566098, ENST00000579468, ENST00000444682 were correlated with forced expiratory volume in one second (FEV1)/forced vital capacity (FVC). They also had a close relationship with Th2-associated cytokines that ENST00000566098, ENST00000583179, ENST00000579468 and ENST00000444682 were positively correlated with IL-13. However, ENST00000583179 was negatively correlated with IL-4 and IL-6. ENST00000579468 was positively correlated with IL-5. Also, ENST00000444682 was positively correlated with IL-5 and negatively correlated with IL-6. After constructing the coexpressed lncRNA-mRNA network, these four lncRNAs were seen to be involved with Th2-type immune response and coexpress with genes (SMAD7, WNT2B, C/EBP, T-bet, and NF-kB) in asthma pathogenesis, which contributed to modulate Th2 cell differentiation and the related proinflammatory factor production.

### lncRNA Associated With Th17/Treg Imbalance

The imbalance of Th17/Treg has been reported to be mainly involved in asthma pathogenesis, including neutrophil recruitment and exacerbation of airway inflammation mediated by upregulated Th17-type cytokines (IL-17A, IL-17F, and IL-22) and downregulated Treg-type cytokines (IL-10, and transforming growth factor (TGF) -β) (Newcomb and Peebles, 2013; Boonpiyathad et al., 2020). Th17 cells exert the proinflammatory function while Treg cells avoid the immune response overactivation and function immune tolerance. Th17-type inflammation is always accomplished with Th1-type inflammation, bringing about neutrophilic, severe, or glucocorticoid-resistant asthma. It was found that IL-17A, IL-17F and IL-22 were increased in peripheral blood mononuclear cells (PBMCs), bronchoalveolar lavage fluid (BALF) and sputum of asthmatic patients, mainly in those with severe asthma (Doe et al., 2010; Chesne et al., 2014; Raedler et al., 2015). Furthermore, IL-17/IL-10 ratio was remarkably enhanced in the patients of asthma exacerbation, which implied that Th17 cells and Treg cells didn’t keep balance (Zou et al., 2018). Currently, the studies have focused more on the role of Th17/Treg balance and would like to explore the novel treatments targeting IL-17A, IL-17F or IL-22. It has been confirmed that IL-17 or IL-22 deficient mice have lower proinflammatory factors levels and the alleviation of airway inflammation (Yang et al., 2008; Lilly et al., 2012). Brodalumab, which targets IL-17Rα, thus blocking both IL-17A and IL-17F, was explored in the clinical but was not found any improvement in the lung function of asthmatic patients, probably due to the participants in this trial who were not selected for neutrophilic inflammation (Busse et al., 2013). On the aspect of lncRNAs in the imbalance of Th17/Treg, Qiu et al. (2019) confirmed that lncRNA MEG3 acted as a ceRNA in the regulation of Th17/Treg balance in asthma pathogenesis. According to the bioinformatic evidence, 19 miRs were selected that had the potential to target Treg-associated transcriptional factor Foxp3 or Th17-associated transcriptional factor RORγt and 25 lncRNAs were selected that interacted with at least one of above the 19 miRs from the differentially expressed miRs and lncRNAs achieved from microarrays. LncRNA MEG3 and miR-17 were chosen for verification and it was indicated that LncRNA MEG3 was increased whereas miR-17 was decreased in CD4^+^T cells of asthmatic patients *via* qRT-PCR, consistent with the microarrays results. With the knockdown and the overexpression of lncRNA MEG3 *in vitro*, lncRNA MEG3 was demonstrated to elevate Th17-associated proinflammatory cytokines (IL-17 and IL-22) and RORγt. According to the further cotransfection, dual-luciferase reporter assay and RNA pull-down assay, lncRNA MEG3 could directly sponge miR-17 as a ceRNA. In addition, miR-17 mimic could decrease the level of IL-17, IL-22, RORγt, and miR-17 could bind to the 3’ UTR of RORγt mRNA. The above findings could be concluded that lncRNA MEG3 functioned as a ceRNA to sponge miR-17, thus repressing the bond of miR-17 to RORγt to prevent RORγt mRNA degradation. The lncRNA MEG3/miR-17/RORγt axis can affect Th17/Treg balance in asthma and provide a powerful basis for clinical treatment.

### lncRNA Associated With EOS Dysfunction

EOS degranulation and recruitment are activated by allergen and develop the aggravation of asthma, especially eosinophilic asthma (Rosenberg et al., 2013; Felton et al., 2014; Lambrecht and Hammad, 2015). EOS act as antigen-presenting cells and activate Th2 cells to amplify allergic asthma (MacKenzie et al., 2001; Trivedi and Lloyd, 2007). Patients with allergic asthma were detected to have EOS elevation in blood, BALF and sputum (Sedgwick et al., 1992; Mesnil et al., 2016), which was also observed in OVA-induced mice (Kung et al., 1994). It has been proposed that the antieosinophilic treatments that target the apoptosis of EOS have antiinflammatory effects (Ilmarinen and Kankaanranta, 2014). The suppression of EOS attenuates airway inflammation induced by dendritic cells in mice (Shen et al., 2008; Jacobsen et al., 2011). Zhu et al. (2018) described that 41 lncRNAs and 271 mRNAs were dysregulated in eosinophilic asthma *via* RNA and lncRNA sequencing. Depending on the constructed lncRNA-mRNA coexpressed network and Pearson’s correlation analysis, lncRNA RP11-401.2 was finally identified to have the highest conformance and stability by qRT-PCR. LncRNA RP11-401.2 was remarkably upregulated *in vivo* in peripheral blood from patients with eosinophilic asthma and further verified by qRT-PCR in their sputum. These results imply that lncRNA RP11-401.2 may be positively associated with the progression of EOS dysfunction and mediate eosinophilic asthma.

### lncRNAs Associated With Macrophage Polarization

Macrophages are the most abundant immune cells in asthma pathogenesis and the associated cytokines, chemokines and transcription factors modulate the progress of asthma (Liu et al., 2014; Saradna et al., 2018). IFN-γ, LPS, and TNF-α activate M1 polarization, linked with Th1 cells, Th17 cells, neutrophilic asthma and severe asthma. IL-4, IL-13, and IL-10 induce M2 polarization, associated with Th2 cells and eosinophilic asthma (Girodet et al., 2016; Shapouri-Moghaddam et al., 2018). Serval researches have proposed that some lncRNAs regulate macrophage polarization in asthma pathogenesis, so we discuss them as follows.

LncRNA AK085865 was illustrated to be aberrant and drive M2 polarization in dermatophagoides farinae protein 1 (Der f1) -induced asthmatic murine (Pei et al., 2020). It was demonstrated that lncRNA AK085865 knockout asthmatic murine had alleviated airway inflammation, less IgE, fewer M2 macrophages and fewer eosinophils. Otherwise, the adoptive infusion of M0 macrophages to the lncRNA AK085865 knockout asthmatic murine resulted in the protection from inflammation exacerbation. LncRNA AK085865 knockout was proved to inhibit M2 polarization to protect the murine from the allergic inflammation. In the further flow cytometry, coculture and the transwell system, M2 macrophages were observed to promote ILC2s differentiation *via* exosomes. These results implied that lncRNA AK085865 had the ability to promote M2 macrophage polarization and then enhance ILC2 differentiation to amplify type 2 inflammation. Notably, the attenuation of inflammation by knockout of lncRNA AK085865 guides the novel treatment in type 2 inflammation. Xia et al. (2020) demonstrated that lnc-BAZ2B had the potential to promote M2 macrophages, which was remarkably upregulated in asthmatic children and mainly expressed in monocytes. In the cockroach allergen extract (CRE) -induced murine, lnc-BAZ2B was found to promote M2 macrophage activation *via* increasing BAZ2B expression, thus aggregating inflammation. In its mechanism, lnc-BAZ2B stabilized BAZ2B pre-mRNA to enhance its cis target BAZ2B and then increased interferon regulatory factor 4 (IRF4) and M2 macrophage activation. With the verification that BAZ2B knockdown led to alleviate asthma severity, BAZ2B was implied to have the ability to act as a target for attenuating asthma. Lnc-BAZ2B knockdown and BAZ2B knockdown are considered to be the possible targets to alleviate type 2 inflammation *via* suppressing M2 macrophage activation. Han et al. (2019) observed that lncRNA PTPRE-AS1 increased the receptor-type tyrosine protein phosphatase ϵ (PTPRE) expression to suppress IL-4-induced M2 macrophages *via* MAPK/ERK 1/2 pathway *in vitro*. *In vivo*, lncRNA PTPRE-AS1 knockout CRE- induced asthmatic murine had increased M2 macrophage activation, worse airway inflammation and the decrease of PTPRE. Mechanistically, the results of the further RNA pull-down and ChIP assays proved that lncRNA PTPRE-AS1 directly bound to WDR5 to modulate H3K4 trimethylation of the PTPRE promoter to repress M2 macrophage activation. Also, it was detected that lncRNA PTPRE-AS1 and PTPRE were reduced in PBMCs from asthmatic patients and the two were positively correlated, which showed the potential of acting as a biomarker for lncRNA PTPRE-AS1 to attenuate M2 macrophage-mediated type 2 inflammation.

## lncRNAs Associated With ASMCs Proliferation in Asthma Pathogenesis

Airway remodeling, an important link in the course of asthma, is closely associated with a wide range of structural and functional cells in the airway, including fibroblast activation, epithelial cell enhancement and ASMCs proliferation (Ai-Muhsen et al., 2011). A few studies have indicated that lncRNAs have a relationship with fibroblasts or epithelial cells. Gu et al. (2020) observed that there was a significant difference in lncRNA KCNQ1OT1 from serum between asthmatic children with airway remodeling and those without airway remodeling. LncRNA KCNQ1OT1 was correlated with the number of fibroblasts and functioned as a predictor of airway remodeling. Wu et al. (2020) confirmed that lncRNA growth arrest specific-5 (GAS5) expressed differentially in PBMCs between nonsevere asthmatic patients and severe asthmatic patients. To further explore its mechanism, it was found that GAS5 could bind to the glucocorticoid receptor and affect its phosphorylation at serine 226 in human bronchial epithelial cells (HBECs) *in vitro*. More importantly, contraction, secretion and proliferation of ASMCs are regarded as the main cause of airway remodeling in asthma (Lazaar and Panettieri, 2005; Lauzon and Martin, 2016). ASMCs phenotype is linked with the activation of the deposition of extracellular matrix proteins and pro-remodeling function of recruited fibrocytes (Mattoli, 2015; Boulet, 2018), exacerbating airway remodeling. It was found that ASMCs proliferation was aggravated in asthmatic patients and there were more ASMCs from asthma subjects than from healthy subjects in post-mortem airway sections (Fujita et al., 2011; Elliot et al., 2018; James et al., 2018). *In vivo*, ASMCs thickening and proliferation were verified to be promoted in house dust mite-induced murine (Yang et al., 2017). Reversely, the diminish of ASMCs could restore bronchial airway thickening of the murine, leading to the relief of airway remodeling (Kohan et al., 2009). Asthma medications that target ASMCs contributed to slow the proliferation and migration of ASMCs to attenuate persistent structural changes in the airway wall (Girodet et al., 2011). A number of studies have already proposed that most lncRNAs function to modulate ASMCs proliferation in asthma pathogenesis, and we review the role of them.

### lncRNA Plasmacytoma Variant Translocation (PVT1)

It was demonstrated that PVT1 was downregulated in ASMCs of glucocorticoid-sensitive nonsevere asthmatic patients and was upregulated in ASMCs of glucocorticoid-insensitive severe asthmatic patients (Austin et al., 2017). TGF-β plus fetal calf serum (FCS) induced ASMCs proliferation. Further in healthy ASMCs, the addition of dexamethasone (DEX) caused an increase of PVT1 to repress ASMCs proliferation, which could be reverted *via* PVT1 siRNA. In severe asthmatic subjects, DEX also caused an increase of PVT1 but PVT1 siRNA induced ASMCs proliferation instead of suppressing the proliferation. Mechanistically, the addition of DEX to severe asthmatic patients enhanced c-MYC expression and PVT1 was a downstream target of transcription factor c-MYC that could be bound by c-MYC to promote ASMCs proliferation. Notably, PVT1 has a great potential to regulate ASMCs proliferation in severe asthma and may act as a predictor of the ASMCs phenotype.

### lncRNA GAS5 (GAS5)

Zhang et al. (2018) observed GAS5 upregulation, miR-10a downregulation and brain-derived neurotrophic factor (BDNF) upregulation in ASMCs isolated from OVA-induced asthmatic rats. *In vitro*, based on the bioinformatics analysis, RNA immunoprecipitation (RIP) and RNA pull-down assay, GAS5 was confirmed to bind to miR-10a. Further in platelet-derived growth factor (PDGF)-BB-induced ASMCs, BDNF was enhanced, which could be reversed by knockdown of GAS5. The inhibition of miR-10a could reverse the inhibitory effect of GAS5 knockdown, which proved that GAS5 bound to miR-10a to enhance BDNF expression. In the validation in asthmatic rats *in vivo*, GAS5 knockdown asthmatic rats had increased miR-10a, decreased BDNF and lower AHR, consistent with the results *in vitro* and showing the antiinflammatory function of GAS5 knockdown. Mechanistically, in the luciferase reporter assay, cotransfection and verification of cell proliferation, PDGF-BB was proposed to promote ASMCs proliferation *via* GAS5/miR-10a/BDNF regulatory axis in asthma. GAS5, which can function as miR-10a’s sponge, binds to miR-10a and affect its binding to BDNF, enhancing BDNF to promote ASMCs proliferation. GAS5 knockdown has the capability to attenuate ASMCs proliferation in asthma, which can be a target to modulate airway remodeling.

### lncRNA TCF7 (lncTCF7)

Upregulation of lncTCF7 and TIMMDC1 was detected in ASMCs isolated from asthmatic patients *via* qRT-PCR (Fan et al., 2019). In MTT assay, EdU assay and transwell assay, lncTCF7 knockdown and lncTCF7 overexpression, lncTCF7 was confirmed to promote ASMCs viability, proliferation and migration to amplify the airway remodeling. Furthermore, TIMMDC1 mRNA and protein and p-AKT were positively correlated with lncTCF7 expression, which could be both facilitated with PDGF-BB treatment and PDGF-BB induced ASMCs proliferation and migration. After conducting the rescue assays, it was concluded that lncTCF7 enhanced ASMCs growth and migration *via* activating the TIMMDC1/AKT signaling pathway, which supplied a potential therapeutic for airway remodeling in asthma.

### lncRNA TUG1 (TUG1)

Lin et al. (Lin J. L. et al., 2019) demonstrated that TUG1 was remarkably upregulated in ASMCs from OVA-induced asthmatic rats *via* RT-PCR. The Th2-associated cytokines (IL-4, IL-5 and IL-13) and IgE were detected to be increased as well. With the inhibition and overexpression of TUG1, CCK-8 assay, transwell assay and flow cytometry, TUG1 was verified to promote ASMCs proliferation and migration. The further bioinformatics analysis, luciferase gene reporter assay and cotransfection revealed that TUG1 acted as a ceRNA to sponge miR-590-5p and prevent its bond to fibroblast growth factor 1 (FGF1), thus promoting proliferation and migration of ASMCs. According to the results above, TUG1 tends to be a therapeutic target for airway remodeling, involved with TUG1/miR-590-5p/FGF1 axis.

### lncRNA Brain Cytoplasmic RNA 1 (BCYRN1)

It was found both BCYRN1 and transient receptor potential 1 (TRPC1) were highly expressed in ASMCs separated from OVA-induced asthmatic rats (Zhang et al., 2016). Overexpression of BCYRN1 promoted the viability, proliferation and migration of ASMCs *in vitro*. Furthermore, after treated by PDGF-BB to induce ASMCs proliferation, the enhancement of BCYRN1 and TRPC1 was further amplified. However, BCYRN1 knockdown reduced ASMCs viability, proliferation and migration, which could be reversed by TRPC1 overexpression. After the further RNA pull-down assay, RIP assay and BCYRN1 overexpression, TRPC1 was proved to be upregulated by BCYRN1 *via* the enhancement of TRPC1 stability. *In vivo*, BCYRN1 knockdown reduced TRPC1, the inspiratory resistance and expiratory resistance. Mechanistically, BCYRN1 increased TRPC1 *via* developing the stability of TRPC1, thus promoting ASMCs proliferation, which provided a novel sight to attenuate abnormal proliferation and migration of ASMCs in asthma.

### LINC00882

Perry et al. (2014) used primary ASMCs with FCS stimulation or DEX combined with FCS treatment *in vitro* and found that LINC00882, LINC00883, PVT1 were differentially expressed *via* microarray and qRT-PCR verification. In further exploration of miR-lncRNA interactions, lncRNAs (LINC00882, LINC00883, BCYRN1, RP11-46A10.4) were predicted to act as sponges of four miRs (miR-371-5p, miR-150, miR-940, miR-1207-5p). Liu Z. F. et al. (2019) further confirmed that LINC00882 was remarkably upregulated in fetal ASMCs induced by PDGF, which enhanced airway remodeling. In the functional experiments with LINC00882 knockdown and overexpression, LINC00882 was shown to promote fetal ASMCs proliferation. Moreover, in the bioinformatics analysis, the luciferase reporter assay and the RNA pull-down assay, LINC00882 was revealed to sponge miR-3619-5p and prevent its bond to β-catenin to enhance PDGF-induced fetal ASMCs proliferation *via* Wnt/β-catenin signaling. The above results indicate that LINC00882 knockdown is probably a novel therapeutic to alleviate airway remodeling in pediatric asthma.

### MALAT1

MALAT1 was reported to have the potential to modulate ASMCs proliferation and migration (Lin L. et al., 2019). *In vitro*, MALAT1 was dramatically upregulated with PDGF-BB stimulation, which promoted the proliferation of ASMCs. The knockdown of MALAT1 led to the inhibition of ASMCs proliferation. Using bioinformatic analysis, MALAT1 knockdown, MALAT1 overexpression, the luciferase reporter assay, the gain and loss of function, MALAT1 was demonstrated to act as a ceRNA for miR-150 and repress its bond to translation initiation factor 4E (eIF4E) to promote ASMCs proliferation *via* Akt signaling. It can be suggested that MALAT1 is potential to be a novel target to modulate airway remodeling in asthma.

## lncRNAs Associated With Glucocorticoid Insensitivity in Asthma Pathogenesis

Glucocorticoid insensitivity is a serious and complicated problem in asthma (Adcock and Ito, 2004; Durham et al., 2011), mainly owing to the defective ligand to the glucocorticoid receptor, abnormal receptor nuclear translocation and abnormal combination with proinflammatory nuclear proteins (Adcock et al., 2008; Barnes and Adcock, 2009; Tonello and Poli, 2009). It has been discussed that lncRNAs, miRs and mRNAs were expressed differentially between the nonsevere glucocorticoid-sensitive asthma and severe glucocorticoid-insensitive asthma (Chen et al., 2019). We show a strong interest in the role of lncRNAs in the regulation of glucocorticoid sensitivity in asthma.

### Cancer Susceptibility Candidate (CASC7)

Liu J. H. et al. (2019) investigated that CASC7 and PTEN were lower while miR-21 was higher in ASMCs from severe asthmatic patients, who responded poorly to DEX treatment. DEX lost its function of attenuating the increase of proinflammatory factors (CCL5, CCL11, and IL-6) and the promotion of phosphorylation of GR in ASMCs in severe asthma, which showed that the glucocorticoid sensitivity was impaired. Combined with cotransfection, bioinformatic analysis and dual-luciferase reporter assays, CASC7 was proved to directly sponge miR-21 and suppress its bond to PTEN, thus enhancing PTEN expression. Additionally, by MTT and ELISA assay, CASC7 elevated GR phosphorylation and the repression of inflammatory factors, enhancing glucocorticoid sensitivity *via* blocking the PI3K/AKT signaling pathway. Taken together, the function of CASC7 provides a novel perspective on elevating glucocorticoid sensitivity in asthma.

### PVT1

We have mentioned in lncRNA plasmacytoma variant translocation (PVT1) that PVT1 regulates ASMCs proliferation in severe asthma. Besides, it mediates glucocorticoid insensitivity in nonsevere asthma (Austin et al., 2017). PVT1 was highly expressed in glucocorticoid-insensitive severe asthmatic patients while it was lowly expressed in glucocorticoid-sensitive nonsevere asthmatic patients. The addition of FCS and TGF-β in ASMCs produced the model of glucocorticoid insensitivity. In nonsevere asthmatic subjects, FCS plus TGF-β increased IL-6. DEX could inhibit the production of IL-6 and increase PVT1 whereas the knockdown of PVT1 reversed the inhibitory effect of DEX, which amplified glucocorticoid insensitivity. These findings implied that PVT1 might regulate glucocorticoid sensitivity, which provided a novel sight on the predict of glucocorticoid effectiveness.

### GAS5

The function of GAS5 to promote ASMCs proliferation has been mentioned in *lncRNA GAS5*. It was also illustrated that GAS5 was closely linked to glucocorticoid insensitivity in airway epithelial cells (AECs) (Keenan et al., 2015). GAS5 was upregulated with either DEX or GR transactivation antagonist and transrepression partial agonist RU486 in BEAS-2B bronchial epithelial cells. GAS5 was increased after treated with proinflammatory cytokines (TNF-α, IL-1α, or TGF-β1), thus inducing the glucocorticoid insensitivity. Alternatively, knockdown of GAS5 by siRNA improved glucocorticoid response and element (GRE) activation, but not restored the airway epithelial glucocorticoid insensitivity induced by TGF-β1. Of note, the knockdown of GAS5 promotes glucocorticoid sensitivity, which is of the great significance of the treatment for those glucocorticoid-insensitive severe asthmatic patients.

## Drugs Potential to Modulate lncRNAs in Asthma

Liao et al. (2020) used the databases to explore lncRNAs which could act as ceRNAs and bound to miRs to regulate mRNAs. They found five significant lncRNAs (MALAT1, MIR17HG, CASC2, MAGI2-AS3, DAPK1-IT1) and eight potential drugs (Tamoxifen, Ruxolitinib, Tretinoin, Quercetin, Dasatinib, Levocarnitine, Niflumic Acid, Glyburide). Moreover, other drugs influence the lncRNA/miR/mRNA axis to attenuate asthma exacerbation, which is described in the following part.

### Schisandrin B and BCYRN1

Schisandrin B (Sch B), isolated from herb Schisandrae, has been demonstrated to relieve oxidative stress, endoplasmic reticulum stress and inflammation in many inflammatory disorders (Leong and Ko, 2015). Zhang et al. (2017) demonstrated that Sch B downregulated BCYRN1 and upregulated miR-150 in both OVA-induced asthmatic rats and mechanical ventilation (MV) -induced ASMCs. Besides, ASMCs activation, proliferation and migration were suppressed by Sch B in MV-induced ASMCs. Based on the cotransfection *via* miR-150 inhibitor, the effects of Sch B were reversed and BCYRN1 was verified to be inhibited by miR-150. Mechanistically, Sch B improved miR-150 and then caused BCYRN1 to diminish to attenuate ASMCs proliferation and migration. Sch B functions as a potential therapeutic for the abnormal ASMCs in asthma.

### α-Asarone and PVT1

α-Asarone is a compound isolated from herb Acorus gramineus (Huang et al., 2013), that has been reported to have antiinflammatory (Kim et al., 2015), antihyperlipidemic and antioxidant effects (Limon et al., 2009). Yu et al. (2017) indicated that α-Asarone inhibited ASMCs activation, proliferation and migration and decreased proinflammatory factors (IL-4, IL-5, IL-13) of BALF in respiratory syncytial virus (RSV) -induced asthmatic rats. In addition, α-Asarone downregulated PVT1, which could exacerbate ASMCs proliferation. With further cotransfection, bioinformatic analysis, RIP assays and dual-luciferase reporter assays, PVT1 was proved to act as a ceRNA to sponge miR-203a, repressing its bond to E2F3 to aggregate ASMCs activation, proliferation and migration. Moreover, α-Asarone relieved ASMCs viability, proliferation and migration *via* the PVT1/miR-203a/E2F3 pathway, which provided a new perspective for the treatment in airway remodeling in asthma.

### Montelukast Sodium and lncRNA PCGEM1 (PCGEM1)

Montelukast sodium is known as an effective drug to treat cough-variant asthma (CVA). Xu et al. (2020) confirmed that this drug reduced inflammatory factor IL-4 and increased antiinflammatory factor IFN-γ to enhance pulmonary function. With the further exploration in OVA-induced asthmatic murine, PCGEM1 was found to be downregulated and overexpressing PCGEM1 amplified the antiinflammatory effects of montelukast sodium, thus enhancing the lung function of the asthmatic murine. The blocking of the NF-κB signaling pathway was proposed to be involved in this process. The specific target of PCGEM1 needed to be explored in the future.

## Conclusion and Perspectives

LncRNAs regulate the balance of proinflammation/antiinflammation *via* the immune cells and associated cytokines. We find that there is no research to discuss the relationship between lncRNAs and Th22, Th9, Th25, or T follicular helper cells, which can be the focus of future research. Additionally, several potential cytokines like TNF-α has been confirmed to be closely linked with lncRNAs in any other inflammatory diseases but not mentioned in asthma. We speculate that they are also of great importance in the regulation of asthma *via* the lncRNA. Besides, lncRNAs contribute to the regulation of airway remodeling and glucocorticoid sensitivity during transcription. Their characteristics make it a potential biomarker for the preclinical identification, diagnosis, prognosis, phenotypes of asthma as well as therapeutic targets. In terms of mechanism, the role of acting as a ceRNA might be a major factor for them to regulate the progress of asthma, which can be predicted *via* bioinformatic analysis and can be further verified *via* the cotransfection, RNA pull-down assay, RIP assay, a dual-luciferase reporter assay, the gain of function experiment and the loss of function experiment. Notably, the functions of lncRNAs are reported to be regulated spatiotemporally. However, the current researches have ignored the time-dependent or space-dependent functions, which might be the highlights of future exploration and validation.

## Author Contributions

XZ contributed conception and design of the study, wrote the manuscript and revised the manuscript. YW and JD contributed to the approval of the revised manuscript.

## Funding

This work was supported by grants from the National Natural Science Foundation of China (Grant No. 81774074 and 81704154), Shanghai science and technology commission (grant no. 17401930300 and 18401971300), Young Elite Scientists Sponsorship Program by China Association for Science and Technology (grant no. 2018QNRC001), and Shanghai science and technology commission scientific research project (Grant 18401901800).

## Conflict of Interest

The authors declare that the research was conducted in the absence of any commercial or financial relationships that could be construed as a potential conflict of interest.
